# A Content Analysis of Web-Based Heat Stress Materials Published by Occupational Health and Safety Ministries, Associations, and Agencies in Canada

**DOI:** 10.1177/10482911241298948

**Published:** 2024-12-19

**Authors:** Emily J. Tetzlaff, Brodie J. Richards, Katie E. Wagar, Roberto C. Harris-Mostert, W. Shane Journeay, Fergus K. O’Connor, Glen P. Kenny

**Affiliations:** 1 70363 Faculty of Health Sciences, University of Ottawa, Ottawa, ON, Canada; 2 Department of Medicine, Dalhousie Medicine New Brunswick, Dalhousie University, Saint John, NB, Canada; 3 Department of Medicine, Division of Physical Medicine and Rehabilitation, University of Toronto, Toronto, ON, Canada; 4 Providence Healthcare - Unity Health Toronto, Toronto, ON, Canada

**Keywords:** risk management, workplace safety, heat stress management, heat strain, heat-illness, climate change

## Abstract

An ever-increasing number of workplaces are becoming heat-exposed due to rising temperature extremes. However, a comprehensive review of Canadian safety materials available to support workplaces in managing this critical hazard has not previously been conducted. We undertook a review and a content analysis of heat stress materials on safety-based ministry, association, and agency websites in Canada (n = 155) to identify content related to heat stress (n = 595). Each document was qualitatively analyzed using NVivo. The most dominant components identified were heat stress control measures (n = 492, 83%), training and education (n = 414, 70%), workplaces and workers at risk (n = 361, 61%), exposure limits and monitoring practices (n = 344, 58%), and emergency response and reporting (n = 249, 42%). However, the content within these programming components was highly variable. While we found that organizations across Canada provide heat stress content, there was evidence of inconsistencies and considerable gaps in the availability of material and the strategies presented to control the critical risk posed by heat.

## Introduction

Heat stress presents a significant occupational health and safety (OHS) risk. Managing occupational heat stress requires a consideration of environmental factors (e.g., temperature, humidity, solar radiation), personal protective equipment (PPE), and work intensity (metabolic heat production),^1^ along with complex workforce considerations (e.g., personal medical factors) and the nature of required work (e.g., production requirements). To manage the hazard posed by heat exposure, on-site OHS managers (e.g., safety coordinators, industrial hygienists, crew chiefs) and front-line workers employ various strategies, such as the application of upper limits for heat stress, and other engineering and administrative controls, hygiene practices, and personal monitoring of physiological strain.^2–4^ However, despite efforts to mitigate risk, workers continue to experience heat-related illnesses (e.g., heat cramps, heat rashes, heat exhaustion, heat stroke),^5–8^ along with psychophysical strain (e.g., discomfort, fatigue), which can exacerbate the risk of traumatic injuries^9,10^ and lead to heat-related labor losses by compromising worker productivity.^11–14^ With an ever-increasing number of workplaces now heat-exposed^6,15^ due to rising global temperatures,^16^ ensuring workplaces are provided adequate guidance on effective heat management is critical.

In Canada, occupational health and safety is regulated by provinces and territories. However, some workforces fall under federal jurisdiction (e.g., airlines, postal services, border services). Currently, there is variability among occupational heat stress regulations in Canada. For example, some provinces and territories have no heat-specific regulations; while some regulate that all sectors must comply with the American Conference of Governmental Industrial Hygienists (ACGIH) heat guidelines; and others only require heat management for particular working environments (e.g., construction or indoor environments), but do not stipulate a specific exposure limit.^17^ Further, agency and safety system structures vary within and between provinces. As a result, workplaces receive guidance from multiple OHS system stakeholders, including provincial/territorial ministries and health and safety associations.

Although differences exist between jurisdictions, the governmental ministries responsible for OHS are generally accountable for creating, implementing, and ensuring compliance with government-mandated legislation and regulations. Various health and safety associations, which are typically funded by the ministries, are then responsible for delivering training and developing workplace tools to support workers across all sectors or those operating in specific sectors (e.g., the Infrastructure Health and Safety Association specifically serves the construction, electrical utilities, aggregates, natural gas, ready-mix concrete, and transportation industries).^18^ OHS managers within individual workplaces use guidance to meet their responsibility to mitigate the adverse effects of occupational hazards by conducting job safety analyses and using them to develop and implement health and safety management programs and policies.^19^ However, in most cases, OHS managers are responsible for recognizing, evaluating, and controlling multiple workplace hazards, of which heat stress is just one.^20^ On top of this, OHS managers and workers play critical roles in monitoring and reporting.^20^ Thus, adequate worker protection from heat requires information and resources that are accurate, accessible, and timely.^21^

To our knowledge, no investigations have directly assessed how OHS managers and workers obtain heat stress information. However, previous studies of safety professionals have demonstrated that they frequently face difficulty finding guidance, or they find an overwhelming amount of information, depending on the safety topic.^21^ Due to the large amount of general safety information available online from safety societies and industry associations,^21^ identifying appropriate and accurate information may prove difficult for OHS managers and workers. For example, recent work has shown that wide variations exist in occupational heat stress programs,^4,22,23^ and that OHS managers and workers consult various sources of guidance when implementing heat stress management programs, including national and provincial safety agencies and provincial safety systems.^4^ However, while it is positive that guidance exists, the contents of these heat-specific safety documents have not been systematically evaluated.

To address these critical knowledge gaps, we undertook a systematized review and a qualitative content analysis of occupational heat stress materials published by OHS ministries, associations, and agencies in Canada to (Objective I) identify what groups currently have heat stress content available and (Objective II) analyze the material to develop an understanding of the content included within the resources. We evaluated the hypotheses that (1) few authorities would have webpages and resources dedicated to occupational heat stress content, and (2) the majority would provide general guidance (i.e., not sector-specific). Additionally, we assessed the hypotheses that (3) most of the content would identify a limited scope of controls and strategies for mitigating occupational heat stress, and (4) few sources would address specific populations at greater risk for adverse outcomes at work.

### Methods

#### Search Strategy

As no database or repository exists of all safety associations within Canada, a broad search of the gray literature was conducted using advanced Google search functions with limits applied by region (i.e., for each Canadian province and territory). A second search was conducted to determine additional safety agencies and sector-specific organizations providing workplace OHS guidance (E.J.T. and K.E.W.). To respect the scope of the analysis and its relevance to Canadian safety legislation, websites were omitted from the study if they were not Canadian. The name and website for each identified association/agency were recorded in an Excel spreadsheet (Microsoft Corporation, version 16).

Three members of the research team (B.J.R., K.E.W., and R.C.H-M.) systematically reviewed the website using the navigation menu and the search function (where available) and a series of pre-identified terms (heat, hot, thermal, environment, and temperature). The content pages of each search result were then systematically reviewed, including all links within secondary pages. When a relevant ‘unit of analysis’ (hereinafter referred to as a document) was located, the information was extracted as a portable document format (PDF). Each record included the location (province/territory), agency name, website URL, document name, document URL, and searcher initials. In addition, the navigation pathway from the landing page to the document was recorded (i.e., Landing page > Employment and Social Development Canada > Reports and Publications: Workplace Health and Safety > Compliance Policy > OHS Compliance, *where > indicates a user-click*). This process was continued until (a) all search terms were tested; (b) the links were only linking back to previously reviewed pages; (c) the links were no longer providing additional heat stress information; and (d) the links guided to other agencies providing heat stress information. In the latter's case, the master list of websites was referenced to ensure the agency identified was included for further investigation.

#### Content Coding

Each document was uploaded to NVivo (v.1.6.2, QSR International, USA). Each file was classified (K.E.W. and E.J.T.) based on province/territory, agency name, document type (information sheets, blog posts, posters, guidance documents, infographics, slide decks), publication date (year/month), target audience, and sector (if applicable). A codebook was then developed with deductive (concept-driven) codes based on five heat stress management programming categories.

*Category 1: Heat Stress Management and Control Measures*: The first set of codes was related to heat stress management and control measures and included (1) workplace controls, (2) personal controls (e.g., alcohol consumption, caffeine, fitness, lifestyle, medication use), and (3) roles and responsibilities in implementing controls. Workplace controls were further categorized based on the hierarchy of controls, including elimination (physically remove the hazard — most effective), substitution (replace the hazard), engineering (isolate people from the hazard), administrative (change how people work), and PPE (protect each person separately with worn equipment — least effective) controls. To note, for this analysis, PPE included both garments used explicitly for their capacity to provide cooling to protect the worker (e.g., remove heavy or wet PPE, such as hard hats or masks during breaks, where appropriate and permitted, to allow for sweat evaporation) as well as any recommendation to modify other existing regulated PPE to reduce radiant heat transfer or the insulative properties of the garment (e.g., transitioning to using light-color workwear).

*Category 2: Heat Stress Education and Training*: The second set of codes was related to heat stress training and education. These codes included the (1) basics of thermoregulation, (2) impacts of occupational heat exposure (e.g., death, health impacts, performance, and productivity), and (3) knowing the signs and symptoms of heat stress.

*Category 3: Workforces and Workers at Risk*: The third set of codes was related to sector-based and individual risk factors related to heat, including (1) workforces at risk (indoor and outdoor) and (2) worker risk factor [acute illness, body size, chronic conditions (e.g., cancer, cardiovascular disease), fitness level, age, experience, acclimation, prior heat-related injury, sex, pregnancy].

*Category 4: Exposure Limits and Heat Monitoring Practices*: The fourth set of codes is related to monitoring practices and included (1) occupational exposure and thermal comfort limits, (2) environmental monitoring (e.g., air quality, air speed, air temperature, humidity, solar load), (3) medical monitoring (e.g., pre-placement medical examinations, on-site medical supervision), (4) physiological monitoring (e.g., heart rate, sweat rate, temperature), and (5) self-monitoring.

*Category 5: Emergency Management and Incident Reporting:* The fifth set of codes captured content related to (1) emergency management and first aid procedures specific to heat, (2) site-level reporting processes for heat-related illnesses, and (3) extreme heat event plans.

In addition to the deductive codes, the codebook also allowed flexibility for inductive codes (data-driven) to emerge where necessary. Once the structure of the pre-defined coding frame was developed, the entire research team reviewed the codes to determine if any residual categories should be identified and amended and to seek agreement on definitions, positive indicators, decision rules, and textual examples. A trial coding exercise was completed on a sample of documents (n = 58) by two independent coders (B.J.R. and R.C.H-M.) to determine if the codebook met the requirements of uni-dimensionality (codes are related to a singular construct), mutual exclusivity (content is coded to only one code), and equal dispersion across the data (majority of text is captured) (Schreier et al., 2014). A coding comparison query in NVivo was also used to ensure the category definitions’ consistency (reliability) and validity. The coding comparison was assessed using Cohen's kappa coefficient, a statistical measure of inter-rater reliability that considers the amount of agreement that could occur through chance. NVivo calculates the kappa coefficient individually for each combination code and source and is interpreted as poor agreement (<0.4), fair to good agreement (0.4–0.75), or excellent (>0.75). The coders achieved a kappa value of 0.77 for this analysis, indicating excellent agreement (NVivo, v.1.6.2, QSR International, USA). All remaining documents (n = 595) were fully coded (B.J.R. and R.C.H-M.). Once complete, two coders reviewed all content captured and performed a secondary coding cycle (B.J.R. and E.J.T.).

#### Content Analysis

After coding was complete, the characteristics of the included documents and extracted data (coded findings) were analyzed using a series of NVivo query functions (e.g., dates of publication, publication type, word frequency). All authors then met to discuss the data and agree on the broader interpretation. Descriptive statistics are also presented for each theme and concept, including count (*n*) and percentage (%), where the percentage is shown about the total dataset (n = 595), unless otherwise stated.

### Results

#### Overview of Website Sources

The search identified 155 agency websites eligible for inclusion. A total of 595 documents with content related to occupational heat exposure were found on these sites. Typically, the agency websites provided an average of four different types of documents per site (median: 1, interquartile range [1,3]), including blog posts/bulletins (n = 246, 41%), information sheets (n = 135, 23%), guidance documents (n = 111, 19%), webpage content (n = 31, 5%), infographics/posters (n = 30, 5%), newsletters (n = 16, 3%), resources (e.g., toolbox talks, safety talks) (n = 10, 2%), slide decks (n = 10, 2%), reports (n = 4, 1%), and tri-fold brochures (n = 2, < 1%). These documents represented primarily multi-sector heat safety content (n = 427, 72%); however, some sector-specific material was also identified, including information specific to critical infrastructure sectors (n = 31, 5%), forestry (n = 21, 4%), mining (n = 17, 3%), and transportation (n = 15, 3%). Typically, the content was directed at a range of audiences, including workers (n = 468, 79%), employers (n = 311, 52%), supervisors (n = 113, 19%), and health and safety specialists (n = 30, 5%). The most significant proportion of the content coded was deemed national (n = 218, 37%) (i.e., related to any operations in Canada), followed by content directed at workplaces within the provinces of Ontario (n = 123, 21%) and British Columbia (n = 62, 10%) (Table 1). The material captured in the search was published between 1986 and 2023, with 48% of the content posted in the last decade (2013–2023). Publications were typically released during the Canadian summer (June–August) (n = 190, 32%).

**Table 1.** Sources of Occupational Heat Health Guidance Documents Identified in the Canada-Wide Search.

Location	Count *N* (%)
Canada	218 (37)
Ontario	123 (21)
Prairie provinces (Manitoba, Alberta, Saskatchewan)	78 (13)
British Columbia	62 (10)
Maritime provinces (Newfoundland and Labrador, Nova Scotia, New Brunswick, Prince Edward Island)	62 (10)
Quebec	39 (7)
Northern territories (Northwest Territories, Yukon, Nunavut)	13 (2)

#### Occupational Heat Stress Content

Within the dataset of occupational heat stress documents analyzed, the most dominant components of workplace heat management were heat stress management and control measures (n = 492, 83%), training and education (n = 414, 70%), workplaces and workers at risk (n = 361, 61%), exposure limits and monitoring practices (n = 344, 58%), and emergency response and reporting (n = 249, 42%). The content-coding for each core component is presented below with quotations and examples from the sampled material to provide evidence and illustrate the identified themes and concepts.

#### Category 1: Heat Stress Management and Control Measures

##### Workplace Controls

At least one occupational heat stress control was recommended in 79% of the documents (n = 469, Table 2). The most frequently discussed controls to help minimize the impact of heat on workers were administrative (n = 431, 72%). This included recommendations to implement hydration programs (e.g., water available at the job site, more frequent fluid replacement), work-to-rest regimes, prescribing use of cool break areas, employing alternative time-of-day task scheduling (e.g., strenuous work at cooler times), implementing acclimatization protocols, and establishing programs for employees performing hot work in isolation (e.g., ‘buddy system’). Although less common, a few documents also discussed modifying physical workload during high heat periods (e.g., light tasks only), providing staff with electrolyte replacement beverages and freezies (freeze/ice pop), modifying task pacing, rotating workers through tasks more frequently, reducing the duration of work, and scheduling additional staff.

**Table 2.** Control Measures Referenced Within the Heat Stress Guidance Documents.

Hierarchy of controls	Recommended controls	Count *N* (%)	Textual example of recommended control
Elimination and substitution controls (n = 159, 27%)	Heat hazard elimination	150 (25)	*“**Remove** unnecessary heat sources…Perform work on or near heat sources such as boilers and furnaces when they are not operational.”*
	Remote operation or relocated work	22 (4)	*“Move or **relocate** work away from direct sunlight or radiant heat sources, where possible.”*
Engineering controls (n = 307, 52%)	Shading	222 (37)	*“Determine if some or all of the work can be done in the **shade**.”*
	Air conditioning	157 (26)	*“Have shaded areas, vehicles, or dressing stations with **air conditioning** available.”*
	Fans	108 (18)	*“Use **fans** to increase air movement and help encourage sweat evaporation. Note! This control method is only effective when the air temperature is less than the skin temperature (about 35°C).”*
	Ventilation	97 (16)	*“Improve air temperature through local or general **ventilation**.”*
	Shielding	64 (11)	*“Reduce the radiant heat by placing reflective **shields**.”*
	Mechanical aid (reduce workload)	61 (10)	*“Reduce the level of physical activity required. Reducing heavy physical activity will lower the body's metabolic heat production and, thus, the risk of heat stress. Some examples include using **carts**, **conveyors**, or **mechanical lifting devices**.”*
	Insulation of hot surfaces	49 (8)	*“Control the heat at its source through the use of **insulation** and reflective barriers (*e.g., *insulate furnace walls).”*
	Humidity control	29 (5)	*“Use of **dehumidifier** and elimination of open water baths and steam leaks reduces relative humidity and thus reduces the risk of heat stress.”*
Administrative controls (n = 431, 72%)	Hydration programs	368 (62)	*“Drink one cup of **water** every fifteen minutes.”*
	Work-to-rest	264 (44)	*“Strict **work/rest schedules** must be carefully followed to prevent heat stress. Frequent rest breaks will be needed depending on the working conditions.”*
	Break areas	225 (38)	*“Heat stress and burn hazards are potential problems…cool lunchrooms or **break areas** should be provided.”*
	Task scheduling	218 (37)	*“**Shift operational hours** (*e.g., *film exterior scenes early morning).”*
	Acclimatization protocol	150 (25)	*“Minimize physical activity and allow an adjustment period to **acclimatize** in hot environments.”*
	Isolated worker Protocol	146 (25)	*“Avoid **working alone** in conditions where heat stress is possible – work in pairs, establish a check-in system.”*
	Workload Management	95 (16)	*“Reduce the physical effort needed for the task by…substituting **light tasks** for heavy ones.”*
	Electrolyte replacement	94 (16)	*“Provide potable water and **electrolyte replacement** drinks.”*
	Task pacing	82 (14)	*“Decrease in metabolic heat production through the reduction in workload. Metabolic heat may be decreased by reducing the work **pace**…”*
	Job rotations	59 (10)	*“**Rotate** staff if necessary to avoid physical or heat-related distress.”*
	Modify duration of work	54 (9)	*“Physical demand of the job should be reduced by decreasing the **shift length**.”*
	Additional staffing	37 (6)	*“Reduce the physical effort needed for the task by…increasing the **number of staff** so that more workers share the workload.”*
Personal protective equipment (PPE) (n = 364, 61%)	Light-colored clothing	119 (20)	*“Prevent heat exhaustion by wearing lightweight, **light-coloured**, loose-fitting **clothing**.”*
	General personal protective equipment	77 (13)	*“In some cases, **PPE** can increase the likelihood of hazards such as heat stress.”*
	Evaporative garments	75 (13)	*“Wool clothing can help to minimize heat stress for work near radiant heat sources…Wool clothing deflects radiant heat away from the skin while allowing sweat to **evaporate**. In very hot climates with a lot of direct sun exposure, outdoor workers often wear wool hats to keep cool.”*
	Impermeable clothing	62 (10)	*“Evaporation from perspiration from the skin is the main way the body cools itself. Clothing such as **water-vapour-impermeable, air-impermeable, thermal insulation** or in multiple layers will greatly restrict this heat removal process. The result can be excessive heat strain even when environmental conditions are not hot.”*
	Passive change materials (e.g., cooling vests)	49 (8)	*“Provide the use of jackets or vests with reusable ice packs or **phase change cooling packs** in the pockets.”*
	Reflective clothing	32 (5)	*“The last layer of protection is PPE and the correct clothing such as **reflective** or cooling clothing.”*
	Circulating air	31 (5)	*“Consider offering protective clothing that provides cooling, such as **air-cooled suits** or ice-cooled vests, where practical.”*
	Liquid-cooled garments	30 (5)	*“Vests with the ability to **circulate a coolant** may be considered.”*
	Remove or loosen personal protective equipment	25 (4)	*“**Remove heavy** or wet **personal protective equipment** such as hard hats or masks during breaks, where appropriate and permitted, to allow for sweat evaporation.”* ^a^
	Wetted clothing	21 (3)	*“**Wetted clothing** like terry cloth coveralls that are especially effective in cooling the body when worn underneath reflective and other impermeable protective clothing.”* ^a^
	Facemasks	15 (3)	*“Ill-fitting personal protective equipment such as **masks**, vests, and clothing could create a microclimate around the skin by holding excess heat, moisture and inhibiting sweat evaporation.”*

a Wetting of clothing or removal of personal protective equipment (PPE) specifically discussed concerning emergency response/first aid procedures was coded separately (Table 9).

The second most common control category was PPE (n = 364, 61%), with many sites discussing the importance of considering mandated garment requirements when formulating a heat stress plan. This included suggestions for light-colored workwear. Many documents also provided recommendations for wearable cooling garments, such as evaporative clothing, garments with phase change materials, and circulating air or liquid cooling. In workplaces with radiant heat, the use of reflective clothing (or anti-radiant heat clothing) was recommended. A few documents also recommended employees wet their work clothing to help manage the heat. Lastly, and primarily related to content on managing the COVID-19 pandemic in the workplace, a few documents also discussed the selection of facemasks (e.g., type, material, color) in the context of heat management (i.e., thermal comfort).

Engineering controls (n = 307, 52%) were the third most common strategies discussed in the documents. Recommended strategies were suggested for application during work and rest periods. They included shading workers from the heat, providing air-conditioned spaces for workers to operate in (i.e., buildings or vehicles), and utilizing fans to increase air movement and promote sweat evaporation. Some documents discussed ensuring adequate ventilation in the work areas to reduce heat exposure, shielding or insulating workers from radiant heat where applicable or using mechanical aids to help reduce workload. Lastly, although less common, a few documents discussed dehumidifiers and other strategies to control humidity in the workspace.

Elimination (n = 150, 25%) and substitution (n = 22, 4%) were also recommended for heat management; however, they were used less frequently. This included strategies to directly remove heat production, remove workers from being exposed, or provide a less hazardous alternative (e.g., equipment that produces less heat).

##### Worker Behavior-Based Controls

At least one behavior-based heat stress management control (i.e., adjustable by worker behavior) was identified within 27% of the documents (n = 160) (Table 3). The most common behavior-based heat stress management controls discussed within the documents were avoiding or reducing caffeine intake (n = 110, 19%), avoiding alcohol consumption (n = 107, 18%), modifying nutrition (e.g., smaller portions, increased salt, more carbohydrates) (n = 72, 12%), avoiding sugary beverages (n = 29, 5%), fitness (n = 20, 3%), getting adequate sleep (n = 17, 3%), and stress management (n = 1, <1%).

**Table 3.** Behavioral Control Measures Referenced Within the Heat Stress Guidance Documents.

Recommended controls	Count *N* (%)	Textual example of recommended control
Reduce caffeine intake	110 (19)	*“Drinks with alcohol and **caffeine** should never be taken, as they dehydrate the body.”*
Alcohol consumption	107 (18)	*“There are things that workers can do to prevent heat stress. Like many other health issues, it starts with lifestyle. Being fit, eating properly, getting enough sleep, and **avoiding excessive alcohol** or caffeine will all help your body cope with heat.”*
Meals and nutrition	72 (12)	*“During exercise and work in the heat, your body utilizes more carbohydrates than normal. This means that adding more carbohydrates to your **diet** can improve your performance in the heat.”*
Avoid sugary beverages	29 (5)	*“**Sodas, energy drinks** and alcoholic beverages – despite the contention they are ‘mostly water’ – may not cut it hydration-wise.”*
Promotion of fitness and training	20 (3)	*“Employers should also **encourage employees to stay **fit**** and hydrated and maintain general good health.”*
Promote adequate sleep	17 (3)	*“Individuals should also ensure they get **adequate sleep** (approximately 7 h). Both sleep deprivation and shortened sleep can influence body temperature, in addition to cognitive function and alertness temperature gradients can be significantly affected by lack of sleep.”*
Stress management	1 (<1)	*“Prevention of Heat Stroke: Intrinsic factors within our control:…**stress**.”*

#### Roles and Responsibilities

A quarter of the documents discussed the employers’ and supervisors’ roles and responsibilities in preparing safe work practices and employing controls for workers operating in the heat (n = 148, 25%). Responsibilities included assessing tasks, activities, and occupations where there is a potential for heat stress, evaluating and implementing controls to minimize heat stress, providing training and education regarding heat stress (including identifying/recognizing signs and symptoms of heat stress), maintaining records, and ensuring adequate emergency procedures and first aid (e.g., on-site paramedics). In addition to the role of the employer and supervisors, members of Joint Labor-Management Health and Safety Committees (JHSC) were also discussed in some documents as playing a critical role in heat management. Responsibilities listed included advising the employer on procedures and effective systems to deal with hot environments, supporting the implementation of the heat stress management program, supporting the conduct of heat exposure assessments, promoting and addressing worker concerns and participating in incident investigations. Lastly, a few documents (n = 7, 1%) directly mentioned safety professionals or competent persons who can support the heat management plan in performing measurements and monitoring of ambient conditions and workers.

#### Category 2: Heat Stress Education and Training

##### Basics of Thermoregulation

Descriptions of the body's response to heat were identified in 19% of the documents (n = 112). This typically included explanations of the body attempting to maintain a temperature range between 36 and 38°C, descriptions of the body's heat-regulating mechanisms (e.g., sweat evaporation, dilation of skin blood vessels), as well as how the body generates internal heat or gains heat from the environment (e.g., workload, high temperature).

##### Impacts of Occupational Heat Exposure

The impacts of heat stress were communicated in 50% of documents (n = 299) (Table 4). When discussing the concerns posed by occupational heat exposure, the documents presented three primary categories of impacts. The most communicated impacts were related to immediate (or acute) impacts to health (n = 284, 48%), with the sources typically citing specific heat-related illnesses that workers may experience (e.g., heat stroke, heat exhaustion) or in severe cases— death. The second most common impact area was related to performance, safety, and productivity effects (n = 71, 12%), which typically discussed additional concerns related to accident risk from heat stress or business-related impacts. Lastly, a few sources (n = 48, 8%) also mentioned long-term (or chronic) health impacts that workers may experience, such as cardiovascular issues or organ-specific problems (e.g., liver damage and renal failure).

**Table 4.** References to Short-Term and Long-Term Health, Performance, Safety, and Productivity Impacts of Occupational Heat Stress.

Impact category	Type of impact	Count *N* (%)	Textual example of heat impacts
Immediate (acute) health impacts (n = 284, 48%)	Heat stroke	225 (38)	*“**Heat stroke** – caused by the loss of the body's ability to cool itself through sweating. It is the most serious of the heat stress disorders and requires immediate medical attention.”*
	Heat exhaustion	205 (34)	*“**Heat exhaustion** occurs when the body becomes dehydrated and is unable to regulate its internal temperature.”*
	Death	182 (31)	*“Untreated heat stress can lead to COMA or **DEATH**.”*
	Heat cramps	140 (24)	*“Heat stress is generally broken down into three types – **heat cramps**, heat exhaustion and heat stroke.”*
	Heat rash	120 (20)	*“Heat illness has a range of increasingly serious symptoms, beginning with **heat rash** and muscle cramps, which are warning signs of the onset of more severe heat stress.”*
	Heat syncope	65 (11)	*“Heat exhaustion and **fainting (syncope)** are less serious types of illnesses which are not fatal but interfere with a person's ability to work.”*
	Heat fatigue	22 (4)	*“Heat illnesses include heat rash, **heat fatigue**, heat cramps, heat exhaustion and heat stroke.”*
	Heat edema	18 (3)	*“Heat illness may be viewed as a continuum of diseases relating to the body's inability to cope with heat. It includes minor problems, such as **heat edema** (swelling)…”*
	Non-specific	18 (3)	*“Workers who are unable to cool themselves down may experience symptoms of **heat stress** which, left untreated, may lead to **heat-related illness**.”*
	Heart attacks	10 (2)	*“Left unchecked, heat stress can lead to heat exhaustion, heat stroke, **heart attack**, and other physical health effects.”*
	Musculoskeletal injury (e.g., rhabdomyolysis)	10 (2)	*“When combined with the physical demands of the job, heat stress can contribute to **musculoskeletal injuries** (MSIs).”*
	Hyponatremia	3 (<1)	*“**Hyponatremia** is caused by dehydration and loss of salts and unequal water and salt distribution in the body.”*
	Heat hyperpyrexia	1 (<1)	*“Heat-related illnesses symptoms, prevention and treatment…**heat hyperpyrexia**.”*
Performance, safety and productivity impacts (n = 71, 12%)	Accident risk	64 (9)	*“Heat can contribute to **accidents** in other ways such as the slipperiness of sweaty palms, dizziness, the fogging of safety glasses or respirator facepieces, or trouble concentrating.”*
	Economic losses	10 (2)	*“Left unchecked, heat stress can lead to heat exhaustion, heat stroke, heart attack and other physical health effects. Plus, it can be damaging to business by way of lost **productivity**, disability costs, and fines and penalties.”*
Long-term (chronic) health impacts (n = 48, 8%)	Non-specific	22 (4)	*“Heat illnesses can lead to **long-term health problems** and even death.”*
	Cardiovascular damage (and blood disorders)	18 (3)	*“If heat stroke is not treated immediately, permanent damage to organs (such as the **heart**, brain, kidneys) or even death can occur.”*
	Kidney damage	15 (3)	*“The most serious form of heat stress is heat stroke, which can cause irreversible damage to the heart, lungs, **kidneys** and liver.”*
	Central nervous system damage	12 (2)	*“Heat stroke – this is a medical emergency and requires urgent attention…Most people will have profound **central nervous system** changes…”*
	Liver damage	10 (2)	*“Long-term (chronic) illnesses from heat exposure – some research has shown that certain disorders of the kidney, **liver**, heart,…may be linked to heat exposure.”*
	Coma	9 (2)	*“Untreated heat stress can lead to **coma**.”*
	Reproductive system damage	6 (1)	*“Heat exposure can be associated with temporary **infertility** in both males and females; however, the effects are more pronounced in males.”*
	Gastrointestinal damage	3 (<1)	*“Chronic heat exhaustion has psychological health effects and can cause increased risk for kidney stones and **gastrointestinal diseases**.”*
	Respiratory damage	3 (<1)	*“The most serious disorder is heat stroke, which can cause irreversible damage to the heart, **lungs**, kidneys and liver.”*

##### Knowing the Signs and Symptoms of Heat Stress

Within the source documents, 41% (n = 241) included content on the signs (i.e., observable by co-workers) and symptoms (i.e., discernible only by the worker who may be suffering from heat stress) of heat stress to emphasize the importance of early recognition for the prevention of long-term and more severe health effects (e.g., heat-related illnesses). Within the documents, 170 different variations of signs and symptoms were identified (Table 5). In addition to directly providing information on recognizing the signs and symptoms of heat stress, 44% of the materials also discussed the importance of including this content within employee heat stress training programs (n = 262). For example, statements like “*ensure workers are adequately educated and trained in recognizing the signs and symptoms of heat-related disorders*” were frequently identified. Such statements were commonly accompanied by content indicating the importance of monitoring oneself and peers because “*people suffering from heat stress don’t always recognize their own symptoms*.” Although much less common, 2% of the documents also suggested posting signs related to heat stress awareness (n = 12), such as “*ensure that heat stress warning signs are posted in indoor work areas where workers could be exposed to heat exceeding heat exposure limits*.”

**Table 5.** Signs and Symptoms of Heat Stress Identified in the Occupational Heat Stress Sources.

Hydration and urination	Temperature	Breathing	Swelling
*Urine* Concentrated urine Dark-colored urine Dark yellow to orange urine Less frequent urination Little urine Not passing urine Strong odor of urine *Hydration* Dry mouth Dry tongue Sticky saliva Thirst (feeling thirsty, intense thirst) *Water loss* Body weight loss Excessive weight loss Sunken eyes	Body temperature over 38°C–41°C Chills Goosebumps Low-grade fever Slightly elevated body temperature Very high temperature	Breathlessness (trouble catching breath) Fast, shallow breathing Increased respiration rate Noisy breathing Panting Rapid shallow breathing Trouble breathing (changes to breathing)	Heat edema (swelling) Numbness of the hands or feet Swelling of soft tissue

Mood-based changes	Skin	Muscles	Cardiovascular Complications
*Mood* Anger Agitation Acting strangely Change in mental state Change to personality Depression Have a fit Hostile Irritability (unaccountable) Irrational behaviors Mood change Mood (confused to very angry) Restlessness Seemingly intoxicated behavior Trouble focusing Weird behavior (as if on drugs) *Cognitive functioning* Altered judgement Confusion Decreased level of awareness Decreased alertness Impaired vigilance Loss of ability to do mental tasks Lack of motivation More work errors Problems with memory Problems with reaction time Trouble focusing *General* Fatigue Feeling not well Flu-like symptoms General sickness Headaches Impaired functioning of brain Lethargy Loss of energy Malaise Tired Weariness	*Color* Ashen appearance Grayish skin Pale, clammy skin Pale or bluish skin color Pale or flushed complexion Pale skin tone Red face Reddened skin in the affected area Skin looks muddy or flushed *Wetness* Cold/wet, clammy skin Dry mouth, skin, lips, and eyes Hot, dry skin Persistently damp Skin goes from feeling cold and clammy to hot and dry Skin is moist *Rash* Cluster of pimples or small blisters Heat rash (prickly heat) Miliaria rubra Peeling skin Red blotches Red bumpy rash Skin eruptions Tiny red spots on the skin *Sensations* Pain and itching Prickling sensation Skin may hurt Stinging skin	*Cramps and Spasms* Cramping of muscle (legs, arms, abdomen) Muscle cramps Painful muscle spasms Seizures (convulsions) Sharp muscle cramps *Strength* Difficulty continuing work General weakness Inability to exercise Inability of exertion Lack of strength Loss of ability to do skilled tasks or heavy work Loss of endurance Physical fatigue *Control and coordination* Clumsy Coordination problems Decreased efficiency Loss of balance Loss of muscle coordination and movement Poor muscle control Staggering Uncoordinated *Sensations* Exhaustion Moderate discomfort Tingling and numbness of extremities	Blood pressure drop Changes to pulse rate (strong rapid pulse changes to a weak rapid pulse) Elevated blood pressure Heart rate that does not calm Irregular pulse Normal or low blood pressure Palpitations Pulmonary or cardiac arrests Raised heartbeat Rapid, strong pulse Sustained rapid heart rate
Vision, hearing and speech	Sweating	Gastrointestinal	Consciousness
*Vision* Blurred vision Poor vision Vision problems Visual disturbances Vertigo *Hearing* Blurred hearing *Speech* Incoherent speech Slurred speech	Absence of sweating Dehydration Little to no sweating Profuse sweating Sweating (heavy) Sweating may stop Sweaty palms	Diarrhea Gastrointestinal illness Loss of appetite Nausea (nauseated) Stomach discomfort Stomachache Upset stomach Urge to vomit Vomiting	Collapsing Delirium Disorientation Dizziness (dizzy) Euphoria Fainting (faintness, syncope, parade syncope) Giddiness Hallucinations Lightheadedness Loss of consciousness (partial or complete) Pass out Shock Unresponsive

#### Category 3: Workforces and Workers at Risk

##### Work Force Risk Factors

Overall, 47% of the documents discussed specific heat-exposed sectors (n = 281), including both outdoor (n = 260, 44%) and indoor workforces (n = 145, 24%). For outdoor workplaces, many of the documents provided general statements that stated outdoor workers are at risk (n = 195, 33%) (e.g., “*The sun and warm weather of summer can also bring special hazards for those working outdoors*.”). In addition, 18 other specific outdoor workplaces were identified in the content. The most frequently named industries were construction (n = 57, 10%), agriculture (n = 32, 5%), and landscaping (n = 17, 3%). Similarly, for indoor workplaces, many of the documents provided general statements of risk for these workers (n = 63, 11%) (e.g., “*Indoor workers are also at risk of heat stress*.”). An additional 14 specific indoor workplaces were also identified. Kitchens/bakeries (n = 67, 11%), manufacturing plants (n = 53, 9%), and underground mines (n = 24, 4%) were the most frequently identified at-risk indoor workplaces. A complete list of the occupations identified is presented in Table 6.

**Table 6.** Workforces Identified as at-Risk for Heat Stress.

	Outdoor workforces (n = 260)	Indoor workforces (n = 145)
Generic workforce statements	Outdoor workers (n = 194)	Indoor workers (n = 63)
Specific workforce statements	Construction workers (n = 57) Agricultural workers (n = 32) Landscapers (n = 17) Recreation service providers (n = 12) Forestry workers (n = 9) Electrical utilities workers (n = 5) Open-pit miners (n = 5) Public service workers (n = 5) Other (e.g., firefighters, law enforcement officials, mail service providers, etc.) (n = 20)	Kitchen and bakery workers (n = 67) Factory and plant workers (n = 55) Underground miners (n = 24) Laundry workers (n = 22) Steel mill and foundry workers (n = 20) Other (e.g., oil and gas facility workers, steam tunnel workers, etc.) (n = 22)

##### Worker Risk Factors

Worker-specific factors that potentially increase the risk of heat-related morbidity and mortality were identified in 32% of the documents (n = 192) (Table 7). The most common risk factors related to heat stress discussed in the material were medical conditions (n = 145, 24%). They included generic statements of risk related to medication use (n = 102, 17%), presence of chronic disease (n = 72, 12%) or cardiovascular disease (n = 60, 10%), acute illness (n = 38, 6%), and prior heat injury (n = 34, 6%). The most cited risk factors (n = 119, 20%) were age (n = 114, 19%; older adults n = 94, 16%, young adults n = 29, 5%), body size (n = 61, 10%), and sex/gender (n = 9, 2%). Lack of acclimatization (n = 96, 16%) and work experience (n = 27, 5%) were also identified as occupational risk factors. Lastly, a few documents noted lifestyle risk factors (n = 90, 15%), such as physical fitness level (n = 76, 13%), alcohol/drug use (n = 42, 7%), and sleep deprivation (n = 3, < 1%). It was also noted that 8% of the documents recommended pre-placement examinations to help identify individual-level risk factors (n = 50). For example, “employees should also under [go] a heat tolerance test so that their individual tolerance ability can be noted down in regard to their job.”

**Table 7.** References to Risk Factors for Occupational Heat Stress.

Category	Risk factor	Count *N* (%)	Textual example of risk factor statements
Medical conditions (n = 145, 24%)	Medication use	102 (17)	*“Predisposing factors for heat-related disorders include…use of **medication** that may inhibit sweating, reduce blood flow or cause dehydration (for example antihistamines).”*
	Chronic conditions (generic)	72 (12)	*“Pay attention to workers with special needs, including those with **medical conditions**.”*
	Cardiovascular disease	60 (10)	*“People with **heart disease**…may need to take special precautions.”*
	Acute illnesses	38 (6)	*“People who are used to working in extreme temperatures can have an underlying condition (such as coming down with a **flu** or **cold**) that changes how their body reacts to the temperature.”*
	Prior heat injury	34 (6)	*“Heat stroke occurs more easily when the body has suffered a **previous heat disorder**.”*
	Metabolic disease	28 (5)	*“Predisposing factors for heat-related disorders include…pre-existing medical conditions and treatment (for example, **diabetes**…).”*
	Respiratory disease	26 (4)	*“Workers with pre-existing conditions such as **COPD, asthma**…are at greater risk for heat-related illnesses.”*
	Pregnancy	20 (3)	*“**Pregnancy** is also another factor which plays a role in heat stress management in women, where **pregnant** women are more susceptible to heat-related illnesses.”*
	Skin disease	15 (2)	*“People with **skin diseases** and rashes may be more susceptible to heat.”*
	Physical disability	13 (2)	*“Remember that workers who may be at greater risk (those with medical conditions or **physical impairments**) may need help to stay cool.”*
	Psychiatric illness	10 (2)	*“Hot temperatures can be dangerous, especially if you have…a **mental illness** such as **depression** or **dementia**.”*
	Renal disease	8 (1)	*“Being physically active provides many health benefits, but during extreme heat, it can put you at risk even if you are healthy. Your risk increases if you have:…**kidney problems**.”*
	Cancer	1 (<1)	*“Avoid placing ‘high risk’ employees in hot work environments for extended time period (‘high risk’ individuals are those that suffer from **cancer**…”).*
	Recent vaccination	1 (<1)	*“Other personal factors that can increase a person's risk of heat stress include…recent **vaccinations**.”*
Demographic variables (n = 119, 20%)	Age: Older	94 (16)	*“**Age (particularly for people about 45 years and older**),… makes people more susceptible to feeling the extremes of heat.”*
	Body size	61 (10)	*“Those with **extra weight** often have trouble in both cold and hot situations due to the body having difficulty maintaining a good heat balance.”*
	Age: Younger	29 (5)	*“**Young** men in manual occupations are the most vulnerable to extreme heat and heat illness.”*
	Biological sex or gender	9 (2)	*“**Gender** is an influencing factor since men tend to have a higher sweat rate and larger oxygen intake and therefore tend to acclimatize better than women.”*
Occupational characteristics (n = 103, 17%)	Unacclimatized to work environment	96 (16)	*“Remember that **lack of acclimatization**,… increases susceptibility to heat stress because the body is already in a weakened state.”*
	Work experience	27 (5)	*“If practical, workers in hot environments should be encouraged to set their own work and rest schedules. **Experienced workers** can often judge heat stress and limit their exposure accordingly. **Inexperienced workers** may need special attention as they may continue to work beyond the point at which signs of heat stress begin to appear.”*
Lifestyle factors (n = 90, 15%)	Fitness level	76 (13)	*“Each person is different. People who are in good health and **physically fit** tend to adjust faster and more easily. However, some individuals may not be able to fully acclimatize regardless of their health or physical condition.”*
	Alcohol consumption and drug use	42 (7)	*“Predisposing factors for heat-related disorders include:…**alcohol abuse and recreational drugs**.”*
	Sleep deprivation	3 (<1)	*“**Sleep deprivation** can also increase the risk of heat stress.”*

Abbreviation: COPD, chronic obstructive pulmonary disease.

Note: The content in this table is related to factors that influence susceptibility and vulnerability to heat as a predisposing consideration. In contrast, content about workplaces providing information to employees on improving these behaviors or lifestyle modifications is discussed above in Table 3. For example, alcoholism is a predisposing risk factor for heat-related illnesses (Table 7), whereas promoting coming to work alcohol-free or rehydrating via non-alcohol beverages is a prevention control (Table 3).

#### Category 4: Exposure Limits and Heat Monitoring Practices

##### Occupational Exposure and Thermal Comfort Limits

Specific jurisdictional regulations and guidelines for heat exposure were cited within 21% of the documents (n = 125). Rules from each province or territory were cited at least once. At least one form of occupational exposure limit guidance was mentioned in 18% of the documents (n = 110). Organizations can use these limits when developing their workplace heat stress plans. Cited guidelines included the ACGIH Threshold Limit Values (n = 101, 17%), the National Institute for Occupational Safety and Health (NIOSH) occupational exposure criteria document for working in heat and hot environments (n = 16, 3%), and the Occupational Health Clinics for Ontario Workers (OHCOW) Humidex-based heat response plan (n = 16, 3%). A few of the documents also discussed thermal comfort limits, with some specific references to the use of Humidex to assess the degree of discomfort (n = 10, 2%), which is an index number used by Canadian meteorologists to describe how hot the weather feels to the average person, by combining the effect of heat and humidity. The American Society of Heating Refrigerating and Air-Conditioning Engineers (ASHRAE) standard (55–1922) for thermal comfort and ventilation in indoor offices (n = 9, 1%) and the Canadian Standard (CSA Z412–17) on office ergonomics for acceptable limits for thermal comfort in indoor work environments (n = 4, < 1%) were also cited.

##### Environmental and Physiological Monitoring

Occupational heat stress monitoring was discussed within 63% of the documents (n = 373). These references included a combination of environmental (n = 191, 32%) and physiological metrics (n = 18, 3%) (Table 8). Specific to environmental monitoring, particular parameters were mentioned frequently across the documents, including humidex (n = 97, 16%), air temperature (n = 87, 15%), humidity (n = 83, 14%), wet-bulb globe temperature (n = 76, 13%), sunlight (solar load) (n = 35, 6%), airspeed (n = 28, 5%), and air quality (n = 21, 4%). Physiological monitoring was recommended by fewer documents (n = 18, 3%), which recommended monitoring heart rate (n = 15, 3%), body temperature (n = 11, 2%), sweat rate or body water loss (n = 4, < 1%), along with the use of wearable monitoring devices (n = 1, < 1%). In contrast to the more quantitative environmental and physiological monitoring, subjective strategies were also referenced in various documents (n = 262, 44%), which primarily addressed on-site supervisors or medical attendants visually monitoring (or watching) and self- and peer monitoring for signs and symptoms of heat stress.

**Table 8.** Monitoring Methods Referenced Within the Heat Stress Guidance Documents.

Type of monitoring	Parameter measured	Count *N* (%)	Textual example of monitoring method
Environmental monitoring (n = 191, 32%)	Humidex	97 (16)	*“Know the **humidex** rating–it combines the temperature and humidity to indicate how hot, humid weather feels to the average person.”*
	Air temperature	87 (15)	*“**Air temperature** measured using a regular thermometer, dry bulb or digital thermometer is an acceptable method of heat stress risk assessment if all of the following conditions apply…”*
	Humidity	83 (14)	“Monitor–record hourly temperature and **relative humidity**.”
	Wetbulb globe temperature (WBGT)	76 (13)	*“The **WBGT** is the ‘gold standard’ because it is an indicator of workplace heat stress that factors in the effects of air temperature, humidity, air movement and radiant energy. It provides a single number measure of ‘perceived heat.’”*
	Sunlight (solar load)	35 (6)	*“The UV Index measures **solar UV intensity**, which is not related to temperature. Many different factors contribute to UV levels, including latitude, altitude, air pollutants, cloud, time of year, time of day, etc. You can monitor the UV index by checking your local weather forecast.”*
	Air speed	28 (5)	*“Ensure atmospheric conditions are monitored, such as temperature, humidity, and **wind** may affect the operator.”*
	Air quality	21 (4)	*“Not only should you be monitoring the temperature, but humidity and **smog levels** play a significant role in how much stress is placed on the body.”*
Subjective monitoring (n = 54, 9%)	On-site medical supervision	54 (9)	*“**Observe** workers on an ongoing basis for symptoms of heat stress…Check that foremen are observing workers on an ongoing basis to recognize symptoms of heat stress.”*
	Self and peer monitoring	23 (4)	*“In any work environment with high temperatures, you should **monitor yourself** and **co-workers**, particularly new co-workers, for signs of heat illness, exhaustion or heat stroke.”*
Physiological monitoring (n = 18, 3%)	Heart rate	15 (3)	*“If the **pulse** is greater than 130 beats/minute, the individual should be allowed to rest during the cooling and rehydration phase until the **pulse** rate drops below 100 beats/minute.”*
	Body temperature	11 (2)	*“If heart rate measurements are insufficient for monitoring worker's exposure to heat stress (for instance, in very heavy work), record his/her oral **temperature**. Using a clinical thermometer, record the **temperature** after work but before the worker drinks water. If the oral **temperature** taken under the tongue exceeds 37.6°C, the next work cycle should be shortened by one-third.”*
	Sweat rate (or body water loss)	4 (<1)	*“Recording **fluid balance** should only be performed if heart rate and temperature measurements are insufficient markers for worker's exposure to heat stress. **Body water loss** will be measured by weighing the worker on a scale at the beginning and end of each workday. The worker's weight loss should not exceed 1 ½ % of total body weight in a workday. If a weight loss exceeding this amount is observed, fluid intake must be increased.”*
	Wearable monitoring	1 (<1)	*“The…device warns workers, via haptic vibration, when their **physiology** indicates the danger of heat stress. Managers get an alert via an app when a worker needs an intervention to stay safe.”*

Abbreviations: WBGT, wet bulb globe temperature; UV, ultraviolet.

#### Category 5: Emergency Management and Incident Reporting

##### Emergency Procedures

Thirty percent of the assessed documents included content on heat-related emergencies (n = 177). The most common steps included seeking medical attention via an on-site paramedic or health attendant (e.g., on-site nurse or physician, first-aid trained staff), moving the individual to a cooler area and providing fluids while waiting for off-site medical support to arrive (e.g., 911 paramedics). However, only 3% of the content specifically stated the need for a first aid response system or emergency response plan (n = 18) (e.g., “develop, equip and practice an Emergency Plan that includes response to heat illnesses, knowing work locations and communicating to 911”). All steps identified are presented in order of dominance in Table 9 with textual examples.

**Table 9.** Steps Identified for Managing Heat-Related Emergencies on-Site.

Action	Count *N* (%)	Textual example of heat impacts
Medical attention	164 (28)	*“Get **medical help** or bring the person to a medical facility.”*
Move to a cooler area	137 (23)	*“Move the person to a **cooler area** where they can rest (such as an air-conditioned building or vehicle or into the shade).”*
Give fluids	124 (21)	*“Give the person **water** to drink (only if they are able to drink it on their own).”*
Remove or loosen excess clothing	101 (17)	*“Take off **excess clothing** (hard hat, boots, shirt, coveralls, etc.).”*
Apply water	88 (15)	*“Remove or loosen tight clothing and **apply cool, wet cloths** such as towels or wet sheets.”*
Fanning	68 (11)	*“Cool the person with cold compresses and **rapid fanning**.”*
Cold compresses	63 (11)	*“Apply cold, wet cloths or **ice** to head, face, neck, armpits, and groin.”*
Salt intake (electrolyte replacement)	53 (9)	*“Provide cool water to drink (**salted** if possible).”*
Lie down	41 (7)	*“**Lie** on back and elevate feet.”*
Rest	35 (6)	*“Fluid replacement and **rest**.”*
Skin hygiene	31 (5)	*“Areas with heat rash should be kept **clean and dry** at all times, and no creams should be applied.”*
Immerse in water	26 (4)	*“Start aggressive cooling with wet cloths, alcohol wipes or **immersion into tepid water**.”*
Massage and stretch	21 (3)	*“**Massage** cramped muscles gently.”*
Stay with the person	21 (3)	*“Call 911 immediately. **Stay with the person** until help arrives.”*
Stop cooling if shivering	5 (<1)	*“**Take care not to cool the worker too much** – if a worker begins to shiver, stop cooling.”*
Monitor temperature	4 (<1)	*“To prevent hypothermia, continue cooling the victim until their **temperature** drops to 102°F (39°C).”*
Maintain airway	3 (<1)	*“Maintain **airway**, breathing, and circulation as required.”*
Elevate head and shoulders	1 (<1)	*“Keep the victim's **head and shoulders slightly elevated**.”*
No medication	1 (<1)	*“You **do not want to provide any medication** whatsoever unless advised by medical staff.”*

##### Heat-Related Illness Reporting

Content on heat-related illness reporting was present within 19% of the source documents (n = 111). Most of this content consisted of simple statements (n = 65, 11%) reminding employees to “report to their supervisor heat stress-related symptoms in themselves or their co-workers” or statements to supervisors ensuring they instruct their staff to “*report if they feel ill*.” However, in some instances (n = 39, 7%), the guidance provided more specific detail on the actions to take for reporting (see Example Box 1).

Box 1. Example of a heat-related illness reporting process.

*Procedure for reporting:*

In case of emergency or if the worker feels ill, the worker should:

— Immediately notify the supervisor, production manager, etc.
— Seek first aid and/or external medical attention when necessary
— Evacuate the area when necessary
— Ensure correction actions are evaluated and/or necessary (for example, if appropriate, shut off the light equipment to reduce the heat)
— Notify appropriate agencies as required

*Investigation:*

The (enter organization name) will conduct an investigation in response to a worker reporting or suffering a heat-related disorder. The following elements will be included in the investigation:

— Description of heat stress problems that have been experienced
— Possible hazards that contributed to and/or caused the condition to occur
— Sources of heat stress in the location

*Exposure Control Plan:*

— Determine whether the incident occurred on a day that was typical of previous weather conditions
— Description of clothing worn by the affected worker
— Confirm whether the worker had been instructed on heat stress, signs and symptoms, and preventive action
— Description of risk controls that had been implemented on the worksite to prevent heat-related disorders
— Evidence of heat exposure measurements/risk assessments being conducted
— Review site documentation, and where appropriate, look for indications of prior heat stress problems

##### Extreme Heat Event (or Hot Weather/Heat Wave) Plans

A few documents (n = 31, 5%) also discussed the importance of having specific plans for extreme heat conditions (i.e., heat waves). These were typically referred to as ‘*hot weather plans*’ or ‘*hot weather action plans*’ and were primarily geared for workplaces that do not usually have hot processes or direct heat exposure.

## Discussion

This study captured the breadth of occupational heat stress resources and guidance available to Canadian workplaces to help them control the critical risks of workplace heat exposure. Contrary to our first hypothesis, many agencies have web pages and resources related to occupational heat stress. However, most materials were either directed at Canadian national audiences or directed specifically at audiences in Ontario or British Columbia. Consistent with our second hypothesis, we found that most guidance was general. It provided limited sector-specific messaging. Additionally, we confirmed our third hypothesis that most of the content identified a limited scope of controls and strategies for mitigating occupational heat stress. As our fourth hypothesis predicted, not much of the material addressed specific populations at greater risk for adverse outcomes at work. Although OHS agencies provide content related to heat stress management (e.g., control measures, training and education, exposure limits and monitoring practices, and emergency response and reporting), there are notable differences in the information provided. Thus, OHS agencies may need to refine guidance that outlines targeted solutions and actions to help provide equitable protection to all workers, especially considering the additional challenges that rising global temperatures will continue to impose.

The Canadian Centre for Occupational Health and Safety (CCOHS) (the federal authority engaged in OHS) should seek to be a leader in revising its heat stress materials, which can serve as a gold standard of heat stress guidance for other jurisdictions. This is especially important for reducing the variability in content across the country and supporting provinces/territories facing resource constraints that limit their capacity for comprehensive content development. The CCOHS’ documentation could be modified by each jurisdiction according to its specific regulations. Further, associations, agencies, and sector-/trade-specific groups embedded within provincial/territorial safety systems should also seek support from their associated ministries to revise or produce tailored heat stress material. For example, in Ontario, the ministry funds six health and safety associations using funding provided by the Workplace Safety and Insurance Board (which is funded by Ontario businesses). As these associations are responsible for the delivery of front-line prevention programs, training and creating tools that can be used in workplaces, they could seek out the allocation of funds for this work.^18^

### Availability of Occupational Heat Stress Material

Our Canada-wide search identified 155 agencies providing nearly 600 documents on occupational heat stress content. Although it may be considered promising that a wealth of material is available, it also raises concern that OHS managers and workers may have access to too many resources, many of which contain incomplete information without a simple, practical method for assessing their quality. This could lead to information fatigue and confusion.^24–26^ Further, despite the vast number of documents available, we found they were not equally distributed across the country. Two provinces accounted for most of the materials identified (Ontario and British Columbia), whereas all other provinces and territories collectively represented only 34% of the materials analyzed. This lack of variability in available materials may be due to the differences in provincial/territorial regulations and OHS system structure across the country. For example, Ontario's OHS system consists of the MLITSD, the Workplace Safety and Insurance Board (an independent agency of the Ministry funded by Ontario businesses), six Ministry-funded health and safety associations (four sector-based and two general), and six research center partners.^18^ In contrast, other provinces have combined Ministry and compensation programs or no direct support associations or research center partners.

Notably, only 48% of the coded content was published in the last decade. This indicates that many organizations hosting these OHS websites may not be archiving dated content or updating content based on the current scientific evidence. There is a critical need to ensure that occupational heat stress guidance is current. Guidance that is no longer reflective of the current state of the literature should be archived to prevent inappropriate reliance on it.

Lastly, we identified that the most common publication period was during the Canadian summer (June-August), raising two concerns. First, although providing content during the summer season may have value because it represents a more apparent priority, it does not give agencies or employers enough time before the temperature rises to prepare, implement, and/or revise controls or to train workers. Guidance should be made available months before the heat season to allow for time to implement it. Secondly, publishing primarily during the summer season may suggest that heat stress is solely environmentally/seasonally driven and thus deemphasizes other critical factors such as heat sources in the workplace, physically demanding work which generates metabolic heat, cumbersome personal protective equipment (e.g., Tyvek suits) which can restrict heat loss or indoor work environments which remain hot, regardless of season (e.g., underground mines, nuclear plants). As it has been well established that heat stress can occur at any time of the year,^6^ and without high-heat conditions present,^27^ organizations need to ensure heat stress is not described as only a seasonal workplace hazard.

### Content of the Heat-Health Material

We found that much of the occupational heat stress material presented management and control measures to mitigate heat stress. However, the two most frequent types of controls represent the least effective strategies within the hierarchy of controls^28^—administrative controls (e.g., hydration programs, work-to-rest regimes) and PPE (e.g., light-colored work wear^29^). The administrative controls most commonly recommended were work practices that reduce the duration, frequency, or intensity of exposure to heat. The emphasis on the bottom of the hierarchy of controls reflects an assumption that it is impossible to eliminate the exposure (e.g., remove equipment that is producing heat entirely) or employ substitution (e.g., alternative equipment which produces less heat) or engineering controls (e.g., shielding, insulating, ventilating) to mitigate risk. This is consistent with the literature on cost. Elimination, substitution, and engineering controls aimed at heat-mitigation tend to be more substantial and costly. However, relying on administrative controls requires a significant, ongoing effort from workers and supervisors and may reduce productivity (e.g., applying work-to-rest regimes based on the environmental conditions and workload may result in less work time).^28^ Therefore, guidance on heat management should emphasize that the controls should always seek to eliminate, reduce, or prevent workers from encountering heat instead of relying on administrative controls and PPE while accepting that existing processes will continue to have heat hazards that are poorly controlled.

There are critical gaps in the current guidance. Only half of the documents described the impacts of heat stress on the body. Of those that did, the priority was short-term health effects (e.g., heat rashes, heat exhaustion) instead of long-term health impacts (e.g., liver damage, renal failure). This is a critical shortcoming because readers (e.g., workers and OHS managers) may underestimate the risk's severity if they are not informed about the long-term health impacts. People, including employers, are more likely to take preventative action if they perceive the health risk to be serious, if they are personally susceptible, and if they understand that the benefits outweigh the costs.^30^ Further, only 12% of the documents discuss the impacts on performance and productivity. This is noteworthy because actual productivity losses due to occupational heat stress at the global level are nearly 10% and are expected to increase up to 30–40% under the worst climate change scenario by the end of the century.^31^ Heat stress can cause substantial productivity losses due to lost work time from heat-related illnesses and fatigue, productivity impairments due to reduced physical and cognitive capacity (i.e., reaction time, accuracy), work output reductions, and healthcare costs related to treatment and rehabilitation, workers’ compensation payments and lost-time injury medical expenses.^31^ If not informed of the performance and productivity impacts of heat stress, workplace management may underestimate or undervalue the potential benefits of implementing controls to prevent heat stress. Employers need to be able to evaluate more accurately the need for heat management and to prioritize it where necessary. To address this, guidance should delineate the adverse short- and long-term health, performance, safety and productivity consequences of heat stress.^6^

Only 41% of the documents included content on the signs (observable by others) and symptoms of heat stress. Within these documents, 170 different terms were used to describe the signs and symptoms of heat stress and some listed signs and symptoms without distinguishing which specific heat-related illness they are indicators of. For example, some documents listed both profuse sweating and the absence of sweating broadly as an indicator of heat stress; however, profuse sweating typically indicates a mild to moderate heat-related illness like heat exhaustion, whereas the absence of sweating can indicate heat stroke. This is important to highlight because the first aid response between the two conditions differ, with heat stroke being a medical emergency due to the potential for a fatal outcome.^32^ As heat stress exists on a continuum, it is critical to emphasize the importance of early recognition, responsiveness, and distinguishing between signs and symptoms of mild and moderate conditions to prevent long-term and more severe health effects.^3,32,33^ We also noted that some terms used as descriptors of heat-related illnesses were unnecessarily complex and should be communicated in plain language to ensure that all readers can understand them (i.e., “heat syncope” replaced with “fainting”).^34^ Therefore, these findings suggest that occupational heat stress guidance should seek to use simple, direct terminology and clearly outline the signs and symptoms of heat-related illnesses, as well as the appropriate emergency response intervention.

Consistent with our second hypothesis, fewer than half of the documents discussed risk factors that place specific workers or workplaces at elevated heat risk. Further, the guidance offers strategies to address these risk factors in very few cases. For example, chronic diseases, such as diabetes, place workers at elevated risk due to disease-related decrements in heat dissipation^35^ as well as altered cardiovascular function and fluid regulation.^36^ As a result of these thermoregulatory impairments, it has been shown that individuals with diabetes have lower tolerance times to prolonged moderate-intensity work in hot environments.^37^ Although the guidance accurately identified individuals with specific risk factors in most cases, some documents failed to provide actionable advice on modifying workplace controls to protect these workers (e.g., providing longer cooling breaks to those with lower tolerance times).

Similarly, some documents identified occupational characteristics that may increase heat susceptibility, including lack of work experience. We highlight this risk factor because current evidence shows that as a worker's job experience increases, the number of heat-related accidents decreases (i.e., traumatic injuries associated with heat-induced syncope and fatigue). For example, an analysis of data from the United States mining industry found that 67% of the heat-related accidents between 2000 and 2017 involved workers with less than 5 years of experience.^38^ Thus, although young workers are more likely to be physiologically responsive to hot environments, as heat loss declines with increasing age^39^ job experience is critical to managing the risks associated with heat exposure in hot and humid workplaces due to factors, such as work efficiency, self-pacing, and familiarity with controls. Therefore, OHS guidance should ensure equitable protection of all workers by providing recommendations to address occupation-specific and individual risk factors.

Our analysis also found that less than a fifth of the documents provided content on heat-related illness reporting and, if present, offered limited guidance on steps or requirements. This is consistent with the widespread under-reporting of occupational injuries and illnesses, including heat stress, across sectors.^40–42^ In addition to the common causes of under-reporting (e.g., fears of retaliation for reporting), various factors have been attributed specifically to the under-reporting of heat-related illnesses and injuries,^10^ including rapid on-site recovery without hospitalization,^43^ misclassification,^20^ and lack of heat-specific claims, programs, or hospital codes^44^ which further complicates the ability to track and discern heat-related illnesses/injuries. As a result, current projections for the Canadian workforce likely underestimate the severity and extent of workers exposed to extreme heat.^45^ Further, the lack of appropriate documentation of potential heat-related injuries may lead to the misguided belief that employees can perform their duties safely when at an elevated risk of a heat-related injury.^4^ Therefore, future guidance should reflect the importance of documenting and providing direction on what should be reported (e.g., environmental conditions, workload, duration of exposure, signs, and symptoms).

Lastly, very few documents (5%) discussed the importance of having specific “hot weather plans” for extreme heat events (heat waves). We highlight this as a critical gap in the current guidance in light of increasing periods of hot weather across Canada^16^ and awareness that extreme heat events can have a wide range of impacts, including directly threatening the health and safety of workers.^15^ Although many heat management strategies employed during general work in the heat can be applied or used during extreme heat events, additional features of a heat event must be considered. For example, extreme heat events are defined by multiple days of consecutive daytime maximum temperatures and nighttime minimal temperatures remaining significantly elevated.^46^ Therefore, workers may not receive a reprieve from the heat to allow themselves to recover adequately and may be increasingly at risk of the heat on consecutive work days.^47,48^

Further, extreme heat events can cause significant elevations in indoor temperatures; thus, workforces operating in indoor environments that may not typically be considered heat exposed (i.e., do not engage in hot work processes) may be at risk if those environments lack air conditioning.^15^ Therefore, guiding the development of hot weather plans is critical to ensure that workplaces are prepared. Guidance should establish:

- an implementation criterion (or thermal trigger) to put the plan into effect (e.g., humidex exceeds a set value or Environment and Climate Change Canada issues a heat warning),
- work procedure changes for periods of elevated temperatures,
- criteria for shutting down operations, and
- communication strategies for providing extreme heat alerts to workers when developing such materials.

### Limitations and Future Studies

This study was limited by the lack of an existing registry of federal or provincial/territorial ministries, safety agencies, and sector-specific organizations providing workplace OHS guidance, specifically on heat stress content. Although we systematically identified relevant associations and agencies across Canada, some may not have been captured in our search. However, having identified 155 agency websites and 595 documents, the findings likely reflect the general state of occupational heat stress risk communication, educational resources, and guidance documents available in Canada. Another limitation of this study is that it did not assess the quality or accuracy of content. As we identified in a previous content analysis of news coverage of the 2021 Heat Dome in Canada, heat stress content often makes critical assumptions about the reader's existing knowledge, provides contradictory messaging, and lacks context for safe application.^49^ Future investigations should evaluate the evidence underpinning guidance to ascertain its accuracy. Further, based on our findings, much of the available content is dated (i.e., only 48% of the content was posted or revised within the last decade; 2013–2023). Future research should examine whether older content contains information that is no longer considered scientifically accurate and/or recommendations that are no longer deemed best practices. Inaccurate, outdated guidance should be removed from the public view.

Additionally, it was not within the scope of this analysis to assess language accessibility, linguistic appropriateness, reading level, or cultural and demographic considerations. Although we did include content reflective of Canada's two national languages, Canadian workers come from many cultures, have many native languages, and reflect various demographic profiles (e.g., genders, age groups, chronic disease groups, etc.). Providing appropriate content in a broader array of languages is essential for protecting all workers. Further, as literacy levels vary within and between heat-exposed occupations, content must also be suitable for the reading grade level of its audience. Future research should investigate the language availability, cultural appropriateness, and reading level of occupational heat stress materials.

Lastly, as identified in our findings, the documents analyzed were published by 155 different safety agencies, government bodies, and industry organizations; thus, the occupational heat stress content varied. However, it was not within the scope of this analysis to assess precisely the factors that may have influenced the variability of content or biased the presentation of the heat stress materials, such as differences in legislation between provinces/territories, union-involvement by sub-sector, or potential political influences (e.g., emphasizing worker behaviors over employer responsibilities). Therefore, to understand fully the intricacies driving the identified variability, further work is necessary that can highlight the potential interests of those who created the materials.

### Practical Applications

Global governing bodies are moving to develop and revise existing legislation, regulations, and support guidance to protect workers from heat stress and heat-related illnesses.^50^ For example, after this study commenced, the Ontario Ministry of Labour, Immigration, Training and Skills Development (MLITSD) proposed a heat-specific regulation within the Ontario Occupational Health and Safety Act (R.S.O. 1990, C.O.1).^51^ The proposed regulation would (a) introduce heat stress exposure limits based on the ACGIH method, (b) provide for the use of other methods to assess a worker's risk of exposure to heat stress, (c) require employers to identify and implement measures and procedures to control heat exposures based on the hierarchy of controls, and (d) require employers to provide worker information and instruction on recognizing the signs and symptoms of heat-related illnesses and the measures to protect themselves.^52^ Within the proposal, the MLITSD also stated that “*Ontario's occupational health and safety system partners provide resources to support Ontario workplaces in identifying and preventing heat stress*.”^52^ However, as our study has demonstrated, various inconsistencies and considerable gaps exist in the current material and strategies presented in the OHS system partners resources. With similar changes on the horizon related to the heat stress policy reform within other Canadian OHS jurisdictions and globally,^50^ climate change fueling more frequent and intense periods of hot weather, and a changing demographic of the workforce (i.e., aging workforce, more women in physically demanding occupations), heat stress management programs are becoming increasingly important for protecting the health and safety of workers.

Therefore, the findings of this investigation (and subsequent work) must be used to ensure that the content provided by these partners is comprehensive, evidence-based, and adequately supports workplaces seeking guidance. We suggest that agencies like the MLITSD develop a best practice or standard for occupational heat stress management to supplement the regulation to ensure workplaces are prepared to comply with the proposed regulation, and safety system partners have the resources to provide comprehensive support. For example, heat stress standards should discuss all validated exposure limit guidelines available (e.g., ACGIH TLVs, Humidex, National Institute for Occupational Safety and Health Recommended Exposure Limits [NIOSH REL], International Organization for Standardization [ISO 7243]) so that workplaces may choose the most feasible and applicable option for their unique context. However, the critical limitations of each guideline and assumptions that must be met in order to apply it properly must also be discussed. The guidance should further specify that workplaces should not alter or modify the guidance tables in any capacity. For example, workplaces should not apply self-directed correction factors when acclimation status is unknown or assume clothing insulation factors when garments are not listed.

Guidance should also be provided on physiological heat monitoring practices, but with abundant caution. First, the guidance should recommend that physiological monitoring be administered only by those with the expertise (e.g., industrial hygienists) to consider both the assumptions and limitations of each method and individual risk factors. Second, guidance on physiological monitoring should not follow a generalized approach, which can be over or under protective. In contrast, this guidance must be individualized and consider factors, such as age, sex, and health status. Third, the standard should also discuss the different ways to conduct physiological monitoring in the field so that workplaces again may choose the most appropriate option. For example, workplaces may consider employing self-monitoring, site-level heat stress indices, measurement of physiological responses to quantify heat strain via wearable technology (e.g., whole-body sweat loss, skin surface temperature, cardiovascular strain), or through the application of subjective assessments (e.g., perceptual strain).^53^ However, each practice has an array of assumptions and critical limitations that, again, must be disclosed. For example, if relying on skin surface temperature measurements to quantify excessive heat strain, considerations must be given to the number of sites of assessment, clothing insulation and permeability, environmental factors, cost, accuracy, and durability in industrial environments, among others.^53^ Lastly, the guidelines must detail the challenges with interpreting these individualized data and the risks of making decisions based on a sole indicator (i.e., wearable technology reporting core body temperature from a field-grade single skin temperature sensor). Further, the guidance must include the requirements for data handling and worker rights for data protection and management.

As with any safety program, ensuring a reporting culture is present is also critical. Therefore, within a newly developed standard, it would be critical to include reporting guidelines for workers and supervisors to record all mild to severe heat-related illnesses and heat-related injuries and accidents. The reporting standard must capture vital components such as environmental conditions (e.g., ambient temperature, humidity, airflow), workwear, work intensity, task duration, and previous experience/exposure to the environment/tasks, among other critical factors. Further, the standard must also detail the appropriate action pathway stemming from reporting (i.e., who is assigned responsibility for determining/applying new controls, how will the worker be kept appraised of the change management process, etc.). Lastly, the standard should propose an enforceable prohibition on policies that intentionally or unintentionally disincentivize reporting.

Comprehensive heat management programming should require heat stress education and training guidelines for all organizational levels, including front-line workers, supervisors, managers, and others. Within these guidelines, it should specify the need for robust training at orientation to work performed in the heat (e.g., new hires, job transfers, migrant workers) or in circumstances where heat stress may occur (e.g., tasks requiring highly insulated PPE), as well as at critical seasonal transitions, and in advance of any additional workplace-specific high-heat periods (e.g., during an operational shutdown where confined space work is completed). This includes providing individuals involved in industrial sector emergency response (e.g., mine rescue, pipe fitters responding to a gas leak) with additional training due to the nature of the work performed that adds to the heat burden (e.g., time-sensitive, continuous, physically demanding work in insulated PPE). Training should consider the signs and symptoms of heat-related illnesses and first aid actions. Additionally, the training should include information on occupational risk factors and present how the organization is controlling and protecting workers from these threats. Further, the inclusion of non-occupational risk factors is also recommended. They include (but are not limited to) factors like alcohol consumption, sun protection, nutrition, and sleep behaviors. Although employers do not have control over the actions of the workforce outside of operational hours, providing education to raise awareness of the rollover effect of one's actions off the job site is critical for heat stress management and worker protection.

## Conclusions

While we identified numerous organizations across Canada providing heat stress content, our review and content analysis provided evidence that inconsistencies and considerable gaps exist in the current availability of material and the strategies presented to control the critical risk posed by heat. Importantly, our study provides insight into the need for guidance to extend beyond surface-level recommendations to include field-based application and specificity by sector and individual-level risk factors. Currently, OHS managers and workers accessing this material online are (in most cases) left to rely on heat stress guidance that was not designed for the unique context of their work environment and workforce demographics and, as a result, lack access to some of the information they need to implement a successful heat stress prevention program. Further, the increasingly unpredictable nature and frequency of extreme heat events exposes more workforces to heat stress. As a result, guidance should address developing hot weather plans. Heat stress policy reform is on the horizon across Canadian jurisdictions. Organizations providing open-access occupational heat stress guidance must provide evidence-driven heat management solutions and guidance that promote the equitable protection of all workers performing their duties in the heat. The knowledge acquired from our study findings will help provide essential recommendations for employers, workers, stakeholders, occupational hygienists, healthcare providers, and others involved in developing guidance and providing control and preventive actions for mitigating occupational heat strain.
